# Increasing Yield of 2,3,5,6-Tetramethylpyrazine in Baijiu Through *Saccharomyces cerevisiae* Metabolic Engineering

**DOI:** 10.3389/fmicb.2020.596306

**Published:** 2020-11-26

**Authors:** Dan-Yao Cui, Ya-Nan Wei, Liang-Cai Lin, Shi-Jia Chen, Peng-Peng Feng, Dong-Guang Xiao, Xue Lin, Cui-Ying Zhang

**Affiliations:** ^1^State Key Laboratory of Food Nutrition and Safety, Key Laboratory of Industrial Fermentation Microbiology, Ministry of Education, Tianjin Industrial Microbiology Key Laboratory, College of Biotechnology, Tianjin University of Science & Technology, Tianjin, China; ^2^Tianjin Engineering Research Center of Microbial Metabolism and Fermentation Process Control, Tianjin, China; ^3^Key Laboratory of Wuliangye-flavor Liquor Solid-state Fermentation, China National Light Industry, Yibin, China

**Keywords:** *Saccharomyces cerevisiae*, 2, 3, 5, 6-tetramethylpyrazine, acetoin, Baijiu, *BDH1*, *BDH2*

## Abstract

Baijiu is a traditional distilled beverage in China with a rich variety of aroma substances. 2,3,5,6-tetramethylpyrazine (TTMP) is an important component in Baijiu and has the function of promoting cardiovascular and cerebrovascular health. During the brewing of Baijiu, the microorganisms in *jiuqu* produce acetoin and then synthesize TTMP, but the yield of TTMP is very low. In this work, 2,3-butanediol dehydrogenase (BDH) coding gene *BDH1* and another *BDH2* gene were deleted or overexpressed to evaluate the effect on the content of acetoin and TTMP in *Saccharomyces cerevisiae*. The results showed that the acetoin synthesis of strain α5-D1B2 was significantly enhanced by disrupting *BDH1* and overexpressing *BDH2*, leading to a 2.6-fold increase of TTMP production up to 10.55 mg/L. To further improve the production level of TTMP, the α-acetolactate synthase (ALS) of the pyruvate decomposition pathway was overexpressed to enhance the synthesis of diacetyl. However, replacing the promoter of the *ILV2* gene with a strong promoter (*PGK1p*) to increase the expression level of the *ILV2* gene did not result in further increased diacetyl, acetoin and TTMP production. Based on these evidences, we constructed the diploid strains AY-SB1 (Δ*BDH1:loxP/*Δ*BDH1:loxP*) and AY-SD1B2 (Δ*BDH1:loxP-PGK1p-BDH2-PGK1t/*Δ*BDH1:loxP-PGK1p-BDH2-PGK1t*) to ensure the fermentation performance of the strain is more stable in Baijiu brewing. The concentration of TTMP in AY-SB1 and AY-SD1B2 was 7.58 and 9.47 mg/L, respectively, which represented a 2.3- and 2.87-fold increase compared to the parental strain. This work provides an example for increasing TTMP production in *S. cerevisiae* by genetic engineering, and highlight a novel method to improve the quality and beneficial health attributes of Baijiu.

## Introduction

Baijiu is an alcoholic beverage that is widely consumed in China (Du et al., 2011). Cereals (sorghum corn, rice, wheat, peas, and millet) and fermentation starter are the main raw materials for brewing Baijiu. The fermentation starter of Baijiu is also known as *jiuqu*, including *daqu*, *xiaoqu*, *fuqu*, and other *jiuqu*. Baijiu brewed from different *jiuqu* will have significant differences in content of aroma and flavor substances. Therefore, Baijiu is classified into five types according to its aroma: strong aroma type, light aroma type, soy sauce aroma type, sweet honey aroma type, and miscellaneous aroma type (Wang et al., 2014). Esters and alcohols are the main flavor substances and their content will affect the quality of Baijiu. Although the kinds of pyrazine are lower than these two volatile flavor compounds, pyrazine is also one of the important aroma compounds in Baijiu (Liu and Sun, 2018). Fan et al. (2007) identified 27 pyrazines in Baijiu by gas chromatography-mass spectrometry (GC-MS), including 2,5-dimethylpyrazine, 2,3,5-trimethylpyrazine, and 2,3,5,6-tetramethylpyrazine (TTMP).

2,3,5,6-Tetramethylpyrazine was originally extracted from the herb of Chuan qiong (*Ligusticum wallichii*), a traditional Chinese medicinal plant (Xiao et al., 2006). TTMP has been previously found to have a beneficial effect on cardiovascular and cerebrovascular health (Xiao et al., 2018; Chen et al., 2020). Moreover, recent studies have shown that TTMP has a positive therapeutic effect on several diseases, including hepatocellular carcinoma and spinal cord injury, and especially with regards to heart toxicity caused by ethanol (Shin et al., 2013; Cao et al., 2015). During Baijiu fermentation, TTMP may be produced via the microbial metabolism and acetoin is the precursor of TTMP (Meng et al., 2016; Xu et al., 2018). First, acetoin is used as a substrate to react with ammonium (or ammonia) to form α-hydroxyimine (Xiao et al., 2014). Subsequently, α-hydroxyimine is converted to 2-amino-3-butanone, which condense to form TTMP spontaneously (Xu et al., 2018; Zhang et al., 2019). It has been reported that the content of TTMP also increased with a gradually increased concentration of acetoin, suggesting that acetoin is a precursor of TTMP (Rizzi, 1988; Chen et al., 2010; Zhu et al., 2010; Meng et al., 2020).

In terms of *Saccharomyces cerevisiae*, pyruvate is converted into α-acetolactate by α-acetolactate synthase (ALS), which is encoded by the *ILV2* gene (*YMR108W*) (Brat et al., 2012). In *S. cerevisiae*,α-acetolactate is decarboxylated into diacetyl spontaneously when the cells are cultured in an aerobic environment (Dulieu and Poncelet, 1999). Diacetyl is then reduced into acetoin by 2,3-butanediol dehydrogenase (BDH), which is encoded by the *BDH1* gene (*YAL060W*) (Ehsani et al., 2009; Kim et al., 2013; Wess et al., 2019). Acetoin is converted into 2,3-butanediol under the control of BDH. The *BDH1* gene plays an important role in the synthesis of acetoin and 2,3-butanediol. The *BDH2* gene (*YAL061W*) is adjacent to *BDH1*, Bdh2p is 51% similar to the Bdh1p (González et al., 2010; Li et al., 2017). The overexpression of the *BDH2* gene improved the tolerance of yeast cells to vanillin (Ishida et al., 2016, 2017). Although the function of BDH in the synthesis of 2,3-butanediol has been extensively studied, its effect on TTMP production has not been reported yet (Kim et al., 2013; Choi et al., 2016; Yang and Zhang, 2018).

**FIGURE 1:**
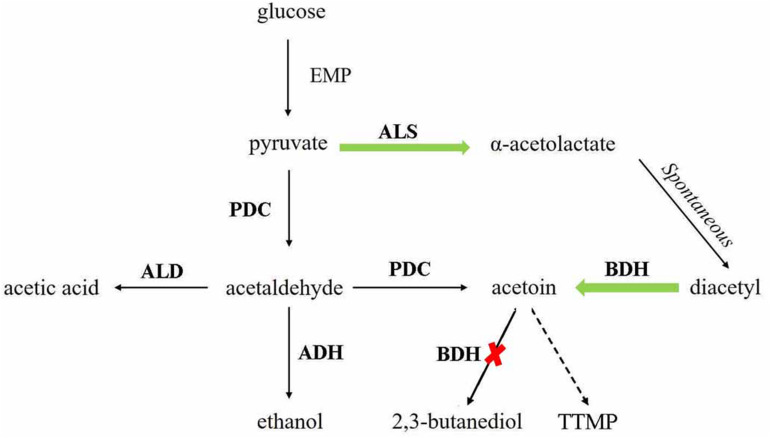
TTMP and acetoin pathway construction in *Saccharomyces cerevisiae.* The *green arrows* indicate the enhanced pathways. The *red cross mark* indicates the disrupted pathways. ALS, α-acetolactate synthase, encoded by *ILV2*; PDC, pyruvate decarboxylase, encoded by *PDC1*, *PDC5*, and *PDC6*; ALD, acetaldehyde dehydrogenase, encoded by *ALD6*; ADH, alcohol dehydrogenase, encoded by *ADH1*, *ADH3*, and *ADH5*; BDH, 2,3-butanediol dehydrogenase, encoded by *BDH1.*

2,3,5,6-Tetramethylpyrazine has been proved to be effective in the treatment of a variety of diseases and beneficial to human health (Hao et al., 2013). However, the content of TTMP in Baijiu is low, so it is interesting to increase the concentration of TTMP in Baijiu. This study is the first to genetically *S. cerevisiae* engineer strains to increase the content of TTMP in Baijiu. The biosynthetic pathway of TTMP and metabolic regulation strategy is shown in Figure 1. In α-type haploid strain, BDH coding gene *BDH1* and another gene *BDH2* were deleted or overexpressed to elucidate their role in TTMP production. Then, recombinant diploid strains were constructed to ensure the fermentation performance during Baijiu brewing. The results demonstrated that overexpression of *BDH2* and deletion of *BDH1* can effectively increase the accumulation of acetoin and then enhance the content of TTMP. The concentrations of ester and higher alcohol produced by the diploid strains were not affected by the genetic modification. In addition, we found that overexpression of the ALS coding gene *ILV2* had no significant effect on the production of acetoin and TTMP. Overall, the diploid strains showed significant potential for industrial applications.

## Materials and Methods

### Strains and Medium

The strains and plasmids used in this study are listed in Table 1. *S. cerevisiae* was grown in YPD medium at 30°C. Then, 1,000 μg/mL G418 (Promega, Madison, WI, United States) was added to the YPD medium to select the right transformants. The YPG medium (10 g/L yeast extract, 20 g/L peptone, 20 g/L galactose) was used to eliminate the *KanMX* resistance gene, as the selection marker for the yeast strain. *Escherichia coli* was grown in LB medium at 37°C. Ampicillin (100 μg/mL) was added to the LB medium as required. The YPD, LB, and YPG mediums were mixed with 2% agar powder for plating.

**TABLE 1:** Strains and plasmids used in the current study.

**Strains or plasmids**	**Relevant characteristic**	**Source**
Strains		
*Escherichia coli*		
DH5α	*sup*E44 Δ*lac*U169 (φ 80*lac*ZΔM15) *hsd*R17 *rec*Al*end*Al*gyr*A96 *thi*1 *rel*A	Stratagene (Santa Clara, CA, United States)
*Saccharomyces cerevisiae*		
AY15	Commercial liquor yeast strain	Tianjin Industrial Microbiology Key Laboratory
a8	*MATa*, haploid yeast strain from AY15	Li W. et al., 2017
α5	*MAT*α, haploid yeast strain from AY15	Li W. et al., 2017
α5-TB1	*MAT*α, Δ*BDH1*:*loxP*	This study
α5-VB1	*MAT*α, Δ*BDH1*:*loxP-PGK1_*p*_-BDH1-PGK1_*t*_*	This study
α5-TB2	*MAT*α, Δ*BDH2*:*loxP*	This study
α5-VB2	*MAT*α, Δ*BDH2*:*loxP-PGK1_*p*_-BDH2-PGK1_*t*_*	This study
α5-D1B2	*MAT*α, Δ*BDH1*:*loxP-PGK1_*p*_*-*BDH2-PGK1_*t*_*	This study
α5-VI2	*MAT*α, Δ*ILV2*:*loxP -PGK1p-ILV2-PGK1t*	This study
AY-B1	*MAT*α*/MATa*, Δ*BDH1*:*loxP*/*BDH1*	This study
AY-SB1	*MAT*α*/MATa*, Δ*BDH1*:*loxP*/Δ*BDH1*:*loxP*	This study
AY-D1B2	*MAT*α*/MATa*, Δ*BDH1*:*loxP-PGK1_*p*_*-*BDH2-PGK1_*t*_*/*BDH1*	This study
AY-SD1B2	*MAT*α*/MATa*, Δ*BDH1*:*loxP-PGK1_*p*_*-*BDH2-PGK1_*t*__/_*Δ*BDH1*:*loxP-PGK1_*p*_*-*BDH2-PGK1_*t*_*	This study
Plasmids		
pUG6	*E. coli/S. cerevisiae* shuttle vector, containing *Amp* marker and *loxP-KanMX-loxP* cassette	Gueldener et al., 2002
Yep352	*URA3* marker, *Amp* marker ori control vector	Invitrogen (Carlsbad, CA, United States)
pSH-Zeocin	*Zeo* marker, Cre expression vector	Li et al., 2017
YEPI2KPB1	*Amp* marker, *Kan* marker, containing *ILV2A*-*loxP-KanMX-loxP−PGK1_*p*_-BDH1-PGK1_*t*_-ILV2B*	Shi et al., 2016
Yep-KPB2	*Amp* marker, *Kan* marker, containing *loxP-KanMX-loxP-PGK1p−BDH2-PGK1t*	Shi et al., 2017

### Construction of Recombinant Strains

The primers used in this study are listed in Supplementary Table 1. The plasmid pUG6 was used to obtain the *loxP-KanMX-loxP* fragment, with the *KanMX* gene used as the selection marker for the yeast strain. To construct the *BDH1* gene deletion strain (α5-TB1) and *BDH2* gene deletion strain (α5-TB2), the plasmid pUG6 was used as the template to obtain the *KanMX-B1* and *KanMX-B2* fragments using the primers B1C-U/B1C-D and B2C-U/B2C-D, respectively. Homologous recombination was used to assemble the fragments into the parental strain α5.

The *ILV2* expression cassette was composed of the homologous sequence fragments of the *ILV2* gene, *ILV2A* and *ILV2B*, the *PGK1* promoter and terminator, the *ILV2* gene, and the *KanMX* gene. The cassette containing the *ILV2* gene was under the control of the *PGK1* promoter and *PGK1p* terminator. The strain α5-VI2 was obtained as follows: the *ILV2A-loxP-KanMX-loxP-PGK1p* and *PGK1t-ILV2B* fragments were obtained through PCR using the primers L1-F/L1-R and L2-F/L2-R, respectively, from the plasmid YEPI2KPB1. The fragment of the *ILV2* gene was obtained using the primers ILV2-F/ILV2-R from the α5 yeast genome.

The construction of the α5-VB1, α5-VB2, and α5-D1B2 strain was similar to that of α5-ILV2. The recombinant fragments and corresponding PCR primers of the constructed strains are listed in Supplementary Table 2. The α5 yeast strain was transformed using lithium acetate/PEG according to the standard protocol (Gietz and Woods, 2002). The transformant cells were grown on a YPD-G418 plate for 48 h at 30°C. The recombinant strains were then verified using PCR with different primers. Finally, the resistance gene *KanMX* of the recombinant strains α5-TB1, α5-VB1, α5-TB2, α5-VB2, α5-D1B2, and α5-VI2 were removed using the Cre/*loxP* procedure.

### Construction of the Diploid Recombinants

To obtain diploid recombinant strains, the a-type and α-type haploid strains were hybridized. These two type strains were cultured in test tubes until the logarithmic growth phase in YPD medium. They were then hybridized in fresh YPD medium for 8 h. The diploid recombinants were tested with MAT-F/MAT-a/MAT-α as primers.

The a-type strain a8 was hybridized with α-type haploid recombinant strain α5-TB1 and α5-D1B2 to obtain the diploid recombinant strains AY-B1 and AY-D1B2, respectively. The *KanMX-B1S* fragment was amplified using PCR with the primers B1C-2U/B1C-2D from the plasmid pUG6. The *B12A-KanMX-PGKp-BDH2-PGKt-B12B* fragment was amplified using the primers B12C-U/B12C-D from the α5-D1B2 strain. The *KanMX-B1S* and *B12A-KanMX-PGK1p-BDH2-PGK1t-B12B* fragments were transferred into the AY-B1 and AY-D1B2 strains to delete the *BDH1* gene of the a8 strain, respectively. The resulting diploid strains were verified by PCR using the corresponding primers. The *KanMX* gene of the resulting diploid strains was eliminated using the Cre/*loxP* procedure, to obtain the AY-SB1 and AY-SD1B2 strains.

### Fermentation Experiments

Corn-semi-solid medium was used to simulate industrial Baijiu fermentation (Li W. et al., 2017). The corn-semi-solid medium was prepared by gelatinizing a mixture of 60 g of corn flour and 130 mL of water at 65°C for 20 min in a 250-mL conical bottle. Then the mixture was liquefied at 90°C for 90 min with thermostable α-amylase (10 U/g corn weight, 2 × 10^5^ U/mL) and subsequently saccharified at 60°C for 30 min with a saccharifying enzyme (150 U/g corn weight, 10 × 10^5^ U/mL). The resulting medium was cooled to 30°C at room temperature. The strains were cultured in a test tube with 8° Bx medium (6 mL) for 24 h. The culture was then added to 12° Bx medium (54 mL) for 16 h. Finally, 15 mL of the strain culture was inoculated into corn-semi-solid fermentation medium for 4 days. The content of residual sugar was measured using Fehling’s reagent.

### GC and HPLC Analysis

The contents of flavor substances (esters, higher alcohols, diacetyl, and TTMP) were detected by gas chromatography (GC) using an Agilent 7890C GC (Agilent, Palo Alto, CA, United States) equipped with an G4513A autosampler, injector, and flame ionization detector (FID) (Cui et al., 2018). HP-INNOWax polyethylene glycol (30 m × 320 μm, i.e., 0.5 μm coating thickness) was used for separation. N-Butyl acetate was used as the internal standard. An internal calibration curve was established utilizing authentic standards to detected the content of the compound. The chemicals were purchased from Merck (Darmstadt, Germany).

High-performance liquid chromatography (HPLC) was used to measure the production of acetoin and 2,3-butanediol. The fermentation broth was diluted to an appropriate concentration and filtered using a 0.22-μm filter. Agilent 1260HPLC equipped with a Bio-Rad HPX-87H column was used, according to the detection method reported by Li P. et al. (2018).

### Real-Time Quantitative PCR

The transcript levels of the *BDH1*, *BDH2*, and *ILV2* genes in the strains were detected using quantitative real-time PCR (RT-qPCR). The *ACT1* gene was used as the reference gene. The primers ACT1-F/ACT1-R, BDH1-F/BDH1-R, BDH2-F/BDH2-R, and ILV2-F/ILV2-R were used to amplify the *ACT1*, *BDH1*, *BDH2*, and *ILV2* genes, respectively. Yeast Processing Reagent (Takara Biotechnol, Dalian, China) was used to extract yeast total RNA, and then reverse transcribed using a PrimeScript^TM^ RT reagent Kit with gDNA Eraser (Perfect Real Time) (Takara Biotechnol, Dalian, China). TB Green PreMix Ex Taq II (Tli RNAseH Plus) (Takara Biotechnol, Dalian, China) was used to detect changes in gene expression levels by RT-qPCR. The PCR amplification program includes 95°C pre-denaturation 30 s, 95°C denaturation 5 s, 60°C annealing polymerization 30 s, melting curve stage 15 s, 60°C 1 min. Real-time PCR was performed using a StepOnePlus real-time PCR system (Applied Biosystems/Thermo Fisher Scientific, Foster City, CA, United States). The 2^–ΔΔCt^ method was used for quantitative analysis.

### Enzyme Assays

The enzyme activities of BDH and ALS were determined as previously described (Calam et al., 2016; Murashchenko et al., 2016). The protein concentration was determined using a TaKaRa Bradford Protein Assay Kit (Takara Biotechnology, Dalian, China). The ALS reaction mixture contained 40 mM pyruvate, 1 mM thiamine diphosphate, 1 mM MgCl_2_, 10 μM FAD, and 100 mM potassium phosphate buffer (pH 7.0). The enzyme activity unit was defined as the amount of acetoin catalyzed by 1 mg enzyme within 1 min under reaction conditions. The BDH reaction mixture contained 10 mM acetoin, 0.1 mM NADH, and 100 mM potassium phosphate buffer (pH 7.0). Acetoin and NADH were used as substrates to measure the activity of BDH. The BDH enzyme unit was defined as 1 μmol of NAD^+^ produced from NADH per min.

### Determination of the Growth Curve

The method used to calculate the growth curve was obtained from Li W. et al. (2018). Briefly, the yeast strain was grown in YPD medium for 12 h up until the stationary phase. Then, the yeast culture was inoculated into fresh YPD medium and cultured in a shaking flask for 24 h. The optical density (OD_600_) was measured every 1 h to obtain the data needed to draw the growth curve.

### Statistical Analysis

Data are provided as the mean ± standard error. The differences between the recombinant strains and host strain were analyzed using Student’s *t*-test. A *p*-value <0.05 was considered statistically significant.

## Results and Discussion

### Effects of *BDH1* Deletion or Overexpression on Production of TTMP in *Saccharomyces cerevisiae*

The *BDH1* gene encodes BDH and plays an important role in the synthesis of 2,3-butanediol and acetoin (Kim et al., 2013). To determine the effect of *BDH1* gene on TTMP production, we constructed *BDH1* deletion strain α5-TB1 and *BDH1* overexpression strain α5-VB1. The TTMP content in the strain α5-TB1 was 1.94-fold higher than that of strain α5 (Table. 2). As the precursor, acetoin production by strain α5-TB1 was 3.77-fold greater than that of α5. Meanwhile, 2,3-butanediol produced by strain α5-TB1 was reduced by 77.33%. The recombinant strain α5-VB1 yielded a 74.85% decrease in acetoin compared to the parental strain. Hence, the production of TTMP by recombinant strain α5-VB1 decreased slightly. The production of 2,3-butanediol by α5-VB1 was increased to 2,702.16 mg/L, which was 1.44-fold greater than that generated by α5. Moreover, deletion or overexpression of *BDH1* gene did not affect the fermentation performance (ethanol production, CO_2_ emission, residual sugar, and growth curve) of the strain (Table. 3 and Supplementary Figure 1A).

**TABLE 2:** The concentrations of TTMP and other metabolites produced by haploid strains.

**Strains^*a*^**	**TTMP (mg/L)**	**Acetoin (mg/L)**	**Diacetyl (mg/L)**	**2,3-Butanediol (mg/L)**
α5	4.04 ± 0.12	601.02 ± 25.23	30.85 ± 1.52	1,866.73 ± 19.96
α5-VI2	4.15 ± 0.15	595.65 ± 35.6	34.06 ± 1.14	1,884.81 ± 24.09
α5-VB1	3.06 ± 0.18	151.1 ± 14.56**	29.41 ± 1.59	2,702.16 ± 37.43**
α5-TB1	7.85 ± 0.12**	2,268.85 ± 50.12**	34.89 ± 2.55**	422.28 ± 13.77**
α5-VB2	5.48 ± 0.12**	1,054.85 ± 26.21**	27.16 ± 0.51**	1,515.81 ± 21.92**
α5-TB2	4.12 ± 0.13	504.38 ± 13.83**	31.341.49	1,667.66 ± 23.36**
α5-D1B2	10.55 ± 0.2**	2,934.53 ± 48.57**	28.37 ± 0.58**	606.21 ± 15.43**

^*a*^Haploid strain: α5 (parental strain), α5-VI2 (*ILV2* overexpression), α5-VB1 (*BDH1* overexpression), α5-TB1 (*BDH1* deletion), α5-VB2 (*BDH2* overexpression), α5-TB2 (*BDH2* deletion), and α5-D1B2 (*BDH1* deletion and *BDH2* overexpression). The strains were fermented by corn-semi-solid medium at 30°C for 4 days. Values are means ± standard deviations from at least three independent tests. Statistical significance is denoted as **P < 0.01 and *P < 0.05.

**TABLE 3:** Fermentation Performances of recombinant strains.

**Strains**	**Weight loss of CO_2_ (g)**	**Ethanol (%, v/v, 20°C)**	**Residual reducing sugars (g/100 mL)**
Haploid strain			
α5	23.3 ± 0.2	15.2 ± 0.1	3.76 ± 0.18
α5-VI2	22.9 ± 0.2	14.9 ± 0.1	3.87 ± 0.12
α5-VB1	23.0 ± 0.3	15.5 ± 0.1	3.87 ± 0.16
α5-TB1	22.8 ± 0.2	15.2 ± 0.1	3.92 ± 0.11
α5-VB2	23.1 ± 0.2	15.0 ± 0.2	4.14 ± 0.15
α5-TB2	22.8 ± 0.3	15.4 ± 0.2	3.71 ± 0.18
α5-D1B2	22.9 ± 0.3	15.1 ± 0.1	3.86 ± 0.12
Diploid strain			
AY15	23.3 ± 0.3	15.4 ± 0.2	3.17 ± 0.12
AY-B1	23.2 ± 0.1	15.2 ± 0.1	3.28 ± 0.19
AY-SB1	23.1 ± 0.4	15.1 ± 0.3	3.26 ± 0.16
AY-D1B2	23.4 ± 0.2	15.6 ± 0.2	2.63 ± 0.22
AY-SD1B2	23.1 ± 0.3	15.2 ± 0.1	3.06 ± 0.14

*The strains were fermented by corn-semi-solid medium at 30°C for 4 days. Values are means ± standard deviations from at least three independent tests.*

**FIGURE 2:**
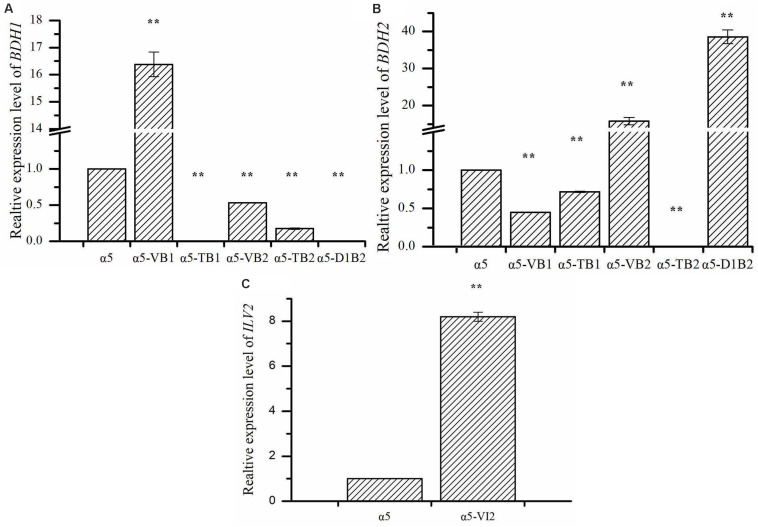
The relative expression levels of *BDH1*, *BDH2*, *and ILV2*. **(A)** The relative expression of *BDH1* in strain α5, α5-VB1, α5-TB1, α5-VB2, α5-TB2, and α5-D1B2. **(B)** The relative expression of *BDH2* in strain α5, α5-VB1, α5-TB1, α5-VB2, α5-TB2, and α5-D1B2. **(C)** The relative expression of *ILV2* in strain α5 and α5-VI2. Data represent the mean of three independent biological replicates. Error bars represent the SD of the average values. Statistical significance is denoted as **P < 0.01 and **P < 0.05.

**FIGURE 3:**
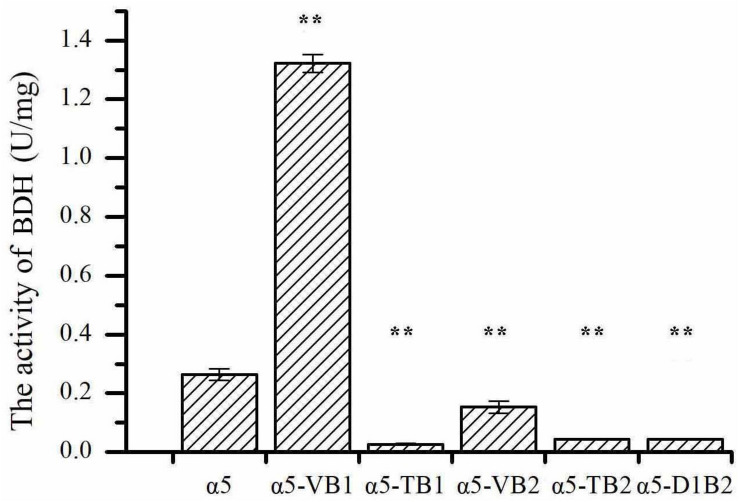
The specific activities of 2,3-butanediol dehydrogenase (BDH). Data represent the mean of three independent biological replicates. Error bars represent the SD of the average values. Statistical significance is denoted as **P < 0.01 and **P < 0.05.

The expression of *BDH1* at the transcriptional level in strains α5-VB1 and α5-TB1 was also measured by RT-qPCR. As shown in Figure 2A, the relative expression of the *BDH1* gene in the recombinant α5-VB1 strain was 16.38-fold that of α5. In strain α5-TB1, the *BDH1* relative expression level was approximately zero. The expression of *BDH2* in α5-VB1 and α5-TB1 were 0.45- and 0.72-fold that of α5. Compared with the parental α5 strain, the level of BDH enzyme activity in the α5-VB1 strain were significantly improved. As shown in Figure 3, the specific activity of BDH in the parental strain α5 at 25°C was 0.264 U/mg. The activity of BDH in the recombinant α5-VB1 and α5-TB1 strains was 1.32 and 0.027 U/mg, respectively. This result is consistent with the transcription assay shown in Figure 2A. In our study, the enzyme activity of BDH was almost reduced to zero after knocking out the *BDH1* gene. This indicates that the *BDH1* gene is the main BDH encoding gene, which supports the conclusion that the expression of *BDH2* gene is not related to BDH activity (González et al., 2001, 2010).

In the present work, *YAL060W* gene was considered to encode BDH, namely *BDH1* (González et al., 2000, 2001). Many subsequent reports focused on the regulating the production of glycerin, isobutanol, and 2,3-butanediol in laboratory strain of *S. cerevisiae* (Ehsani et al., 2009; Kim et al., 2014; Wess et al., 2019). However, the Bdh1p activity of industrial *S. cerevisiae* strain may be different from that of laboratory strain. The results of Kuang et al. (2020) demonstrated that the reduction of acetaldehyde, glycolaldehyde and furfural by Bdh1p from industrial strain was better than that of Bdh1p from laboratory strain. Therefore, we further deleted and overexpressed *BDH1* gene in industrial strain α5, resulting in strain α5-TB1 and α5-VB1, respectively. The production of acetoin indicates that the deletion of *BDH1* gene is beneficial to the synthesis of acetoin. After knocking out the *BDH1* gene, the relative expression level of the *BDH1* gene and the enzyme activity of the BDH decreased, which resulted in the pyruvate flux was successfully redirected toward acetoin pathway (Bae et al., 2016). With the accumulation of acetoin, the TTMP produced during the fermentation of Baijiu has also increased, which is consistent with the previous research conclusions (Hao et al., 2013; Zhang et al., 2019; Zhong et al., 2020).

Many reports have focused on reducing or eliminating the production of glycerol and ethanol by knocking out the *ADH1-5*, *PDC1*, *PDC5*, *GPD1*, and *GPD2* genes, which redirect metabolic flux to 2,3-butanediol or acetoin for high-yield production (Kim et al., 2013; Kim and Hahn, 2015; Bae et al., 2016). However, although the content of acetoin in *S. cerevisiae* strain with *ADH1-5* gene deletion was increased, the yield of ethanol was significantly reduced, which was unfavorable for Baijiu fermentation. Moreover, the simultaneous knockout of *PDC1* and *PDC5* genes would affect the fermentation performance of the engineered strain (Oud et al., 2012). In addition, the introduction of acetoin biosynthetic pathway (ALS and α-acetolactate decarboxylase) from *Bacillus subtilis* into *S. cerevisiae* can also significantly increase the concentration of acetoin (Kim and Hahn, 2015; Jia et al., 2017). However, the insertion of heterogeneous genes may pose a hidden danger to the safety of the strain (Gibson et al., 2018). Therefore, these strategies are not suitable for the construction of high-yield TTMP-producing Baijiu industrial strain.

### Effects of *BDH2* Deletion or Overexpression on Production of TTMP in *Saccharomyces cerevisiae*

The amino acid sequence of Bdh2p is 51% identical to that of Bdh1p, but the effect of Bdh2p on the production of acetoin and TTMP have not been reported yet. We determined the effect of *BDH2* overexpression or deletion on TTMP production in *S. cerevisiae*. The concentration of TTMP in the α5-TB2 (*BDH2* deletion) and α5-VB2 (*BDH2* overexpression) strains was 4.12 and 5.48 mg/L, respectively (Table. 2). The recombinant strain α5-TB2 yielded a 16.07% decrease in acetoin compared with the initial α5 strain. Overexpression of the *BDH2* gene increased the production of acetoin by 1.75-fold compared with the original strain. The production of 2,3-butanediol by the α5-TB2 and α5-VB2 strains was 10.62 and 18.75% decrease compared to that produced by α5, respectively. The deletion or overexpression of *BDH2* gene did not have an impact on fermentation performance in Baijiu fermentation (Table 3 and Supplementary Figure 1A).

The expression of *BDH2* at the transcriptional level in α5-TB2 and α5-VB2 was also measured by RT-qPCR. The *BDH2* relative expression level in α5-TB2 was approximately zero. The expression of *BDH2* in α5-VB2 was 15.84-fold higher than that of α5 (Figure 2B). Under the control of *PGK1p* promoter, the relative expression level of *BDH2* gene was increased significantly. Moreover, the *BDH1* gene expression level and BDH activity were detected in strains α5-TB2 and α5-VB2. As shown in Figure 2A, the relative expression of the *BDH1* gene in the recombinant α5-VB2 and α5-TB2 strains was 0.53- and 0.17-fold that of α5. The BDH enzyme activity was also decreased significantly. Obviously, the decrease of BDH enzyme activity of strain α5-VB2 and α5-TB2 was closely related to the expression level of *BDH1* gene. This means that overexpression or deletion of *BDH2* gene affected the transcription level of *BDH1* gene firstly, and then decreased the enzyme activity of BDH. Therefore, the enzyme activity of BDH depends on the transcription level of *BDH1* gene, and has no obvious relationship with *BDH2* gene.

Many recent reports focus on the use of *BDHs* gene to regulate yeast tolerance to vanillin and acetaldehyde. Ishida et al. (2017) found that *BDH2* gene overexpression improved the tolerance of *S. cerevisiae* to vanillin, and this effect was stronger than that of overexpression of *BDH1*. In reduction of acetaldehyde, the activities of Bdh2p were significantly higher than Bdh1p (Kuang et al., 2020). However, few literatures have reported that the overexpression of *BDH2* gene can improve the synthesis of acetoin and TTMP. In our study, we confirmed that overexpression of *BDH2* could reduce the content of diacetyl, thereby increasing the production of acetoin and TTMP. As shown in Figure 2, we found that knocking out or overexpressing the *BDH1* gene will reduce the expression level of the *BDH2* gene. Similarly, the expression level of *BDH1* gene was also reduced to different degrees in *BDH2* deletion or overexpression strains. In the study of González et al. (2010), the same phenomenon occurred when the *BDH1* or *BDH2* gene was deleted. Due to the changes in the transcription level of *BDH1* and *BDH2* genes, the activity of BDH enzymes was also regulated to different degrees. Based on these data, we speculate that there is a potential association between the Bdh1p and Bdh2p in *S. cerevisiae*. But the functions of Bdh1p and Bdh2p were different: Bdh1p was related to the synthesis of 2,3-butanediol, while Bdh2p was related to the synthesis of acetoin. Under the catalysis of Bdh2p, diacetyl is converted into acetoin, increasing the concentration of TTMP. Hence, the diacetyl content produced by the strain α5-VB2 was lower than that of parental strain α5 and the yield of acetoin increased by 1.74-fold. In contrast, the acetoin production was decreased due to the deletion of *BDH2* gene in the α5-TB2 strain. If the main enzyme that converts diacetyl to acetoin is Bdh1p, then the content of acetoin should be increased, but this is contrary to our experimental results. In our study, the acetoin content produced by strain α5-VB1 (*BDH1* overexpression) decreased by 74.85% compared with the strain α5, while the production of 2,3-butanediol increased significantly. Therefore, the main function of Bdh1p is related to the synthesis of 2,3-butanediol. However, the specific mechanism between Bdh1p and Bdh2p is still unclear and needs further research. It is the first report that overexpression of the *BDH2* gene can increase the concentration of TTMP produced in Baijiu fermentation.

### Increasing Production of TTMP by *BDH1* Deletion and *BDH2* Overexpression in *Saccharomyces cerevisiae*

Based on the results of TTMP productions in the strains α5-TB1, α5-VB1, α5-TB2, and α5-VB2, *BDH1* gene deletion and *BDH2* gene overexpression may improve the content of TTMP. Therefore, we constructed the recombinant strain α5-D1B2. The constructed α5-D1B2 strain with a 4.88-fold increase in acetoin production and a 2.63-fold increase in TTMP titer compared to the parental strain (Table. 2). Owing to the combined action of the *BDH1* deletion and *BDH2* overexpression, the final concentrations of diacetyl and 2,3-butanediol were markedly decreased. The fermentation performance of the α5-D1B2 strain was similar to that of parental strain (Table 3 and Supplementary Figure 1A).

Due to the deletion of *BDH1* gene, the relative expression level of *BDH1* was almost zero (Figure 2A). As shown in Figure 2B, the expression of *BDH2* in α5-D1B2 were 38.56-fold that of α5. The expression level of *BDH2* gene was higher than that of single overexpression of *BDH2* gene (strain α5-VB2). It seems that the expression level of *BDH2* gene is influenced by *BDH1* gene, which confirmed our hypothesis that the functions of Bdh1p and Bdh2p are closely related. These changes demonstrated that the combination of *BDH1* gene deletion and *BDH2* gene overexpression could increase the concentration of TTMP significantly, which was the most effective strategy in this study.

### No Influence of ILV2 Overexpression on Production of TTMP in *Saccharomyces cerevisiae*

In *S. cerevisiae*, α-acetolactate is decarboxylated into diacetyl spontaneously. This means that the strategy of enhancing the “α-acetolactate – diacetyl” pathway by genetic engineering could not be used to increase the content of diacetyl. Since pyruvate produced α-acetolactate under the catalysis of ALS, we focused on enhancing this pathway to increase the content of diacetyl (Espinosa-Cantú et al., 2018). In order to determine the accumulation effect of increasing the expression level of ALS on diacetyl production, thereby increasing the content of acetoin and TTMP, we improved the catalytic reaction of the α-acetolactate biosynthetic pathway by overexpressing the *ILV2* gene, resulting in strain α5-VI2. As shown in Table. 2, the diacetyl content in the parental strain α5 and recombinant strain α5-VI2 was 30.85 and 34.06 mg/L, respectively. Except that the content of diacetyl was slightly increased, no other metabolites were affected by *ILV2* overexpression. As shown in Table 3 and Supplementary Figure 1A, the performances of strain α5-VI2 did not change significantly in comparison to the parental strain α5.

**FIGURE 4:**
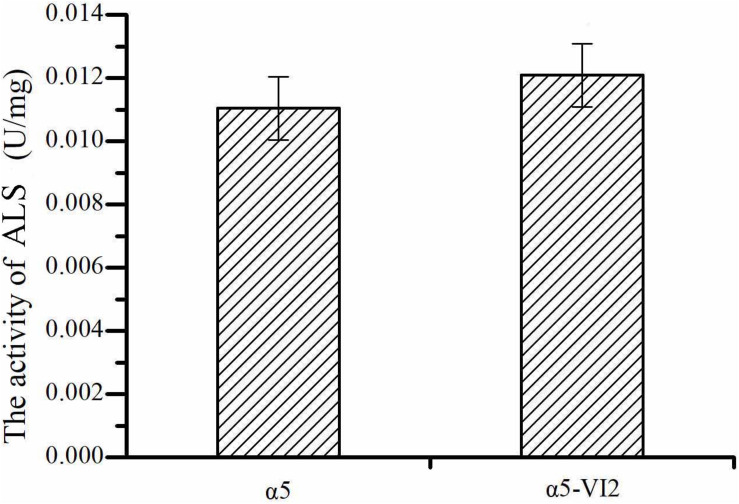
The activities of α-acetolactate synthase (ALS). Data represent the mean of three independent biological replicates. Error bars represent the SD of the average values.

The relative expression level of *ILV2* gene was determined by RT-PCR. As shown in Figure 2C, the relative expression level of *ILV2* was increased significantly, which was 8.21-fold higher than that of the parental strain a5. As shown in Figure 4, the specific activities of ALS in the α5 and α5-VI2 strains were 0.0111 and 0.0121 U/mg, respectively. The overexpression of *ILV2* gene had little effect on the activity of the ALS. Further promoting of α-acetolactate biosynthesis by overexpressing *ILV2* did not significantly improve the concentration of TTMP.

During the fermentation of beer and wine, high concentration of diacetyl was often considered to affect quality of alcoholic beverages (Bartowsky and Henschke, 2004; Humia et al., 2019). In previous studies, we tried to regulate the content of diacetyl by controlling the expression level of *ILV2* gene. Shi et al. (2016) reduced the diacetyl content during beer fermentation by 17.83% by knocking out the *ILV2* gene in brewer’s yeast strain. Meanwhile, the content of diacetyl was decreased by 61.93% in recombinant brewer’s yeast, which deleting *ILV2* gene and overexpressing acetohydroxyacid reductoisomerase encoding gene *ILV5* (Shi et al., 2017). These results indicated that the expression of *ILV2* gene is related to diacetyl biosynthesis. However, in this study, the biosynthesis of diacetyl needs to be improved and then increased the content of acetoin.

The *ILV2* gene overexpression slightly increased diacetyl production, but had no significant influence on the content of TTMP, acetoin, 2,3-butanediol. Although the expression level of the *ILV2* gene increased, it did not significantly affect the activity of the ALS. It has been known that ALS consists of two subunits, the catalytic subunit (Ilv2p) and the regulatory subunit (Ilv6p). A previous study revealed that Ilv2p activity is negatively regulated by the regulatory subunit Ilv6, which may be one of the reasons for affecting the enzyme activity of Ilv2p (Dasari and Kölling, 2011; Takpho et al., 2018). Diacetyl production was also regulated by the expression level of *ILV6* gene. Li P. et al. (2018) reduced the expression level of *ILV6* gene in *Saccharomyces uvarum* by 40% and then diacetyl production was reduced by 25.71%. In lager brewers’ yeast with single and double deletion of *ILV6*, the *ILV6* expression was reduced by 13% and 40%, respectively, and the concentration of diacetyl was significantly reduced. However, the enzyme activity of ALS had not been significantly reduced, which was similar to our experimental results (Duong et al., 2011). It has been reported that the *ILV6* transcription level of native strain with high diacetyl production is higher than that of native strain with low production (Gibson et al., 2015). Meanwhile, overexpression of the *ILV6* gene of *S. cerevisiae* not only enhanced the content of diacetyl, but also increased the transcription level of *ILV2* gene. These results suggest that an effective strategy to increase ALS enzyme activity may be to simultaneously overexpress the genes encoding the regulatory and catalytic subunits, which are *ILV6* and *ILV2* genes.

Moreover, the acetohydroxyacid reductoisomerase (Ilv5p) catalyzes the degradation of α-acetolactate to produce dihydroxy isovalerate, hence the content of diacetyl is also regulated by Ilv5p (Omura, 2008; van Bergen et al., 2016; Shi et al., 2017). In our study, the diacetyl content of strains overexpressing the *ILV2* gene was only slightly increased, which may be related to the decomposition of a part of α-acetolactate by Ilv5p. Furthermore, the conversion of α-acetolactate to diacetyl is a rate-limiting step in *S. cerevisiae* (Kim et al., 2017). This may mean that α-acetolactate needs a large amount of accumulation to significantly increase the concentration of diacetyl. However, α-acetolactate is toxic to *S. cerevisiae* cells and affects the growth performance of the strain (Park and Hahn, 2019). In addition, too high content of diacetyl results in an unwanted buttery flavor, the balance of Baijiu flavor will be broken and the quality of Baijiu will also be decreased. In general, the strategy of promoting the synthesis of acetoin and TTMP by excessively increasing the content of diacetyl is not entirely ideal for Baijiu. Therefore, it is necessary to precisely control the concentration of α-acetolactate and diacetyl to maintain the balance between diacetyl and Baijiu flavor.

### Production of TTMP by Recombinant Diploid Strains

The TTMP content of haploid strains α5-TB1 (*BDH1* deletion) and α5-D1B2 (*BDH1* deletion and *BDH2* overexpression) significantly increased compared with the parental strain α5. In order to investigate the fermentation performance of the strain during Baijiu brewing, four diploid strains (AY-B1, AY-SB1, AY-D1B2, and AY-SD1B2) were constructed based on strains α5-TB1 and α5-D1B2. The production of TTMP and acetoin by diploid strains was investigated by simulating alcohol fermentation experimentally. The data obtained by GC and HPLC are shown in Table. 4. The content of TTMP and acetoin in the parental AY15 strain were 3.29 and 581.13 mg/L, respectively. Compared to the AY15 strain, the AY-B1 strain (*BDH1* single-allele-deletion diploid) yielded a 10.94 and 24.07% increase in TTMP and acetoin production, respectively. The AY-SB1 strain (*BDH1* double-allele-deletion diploid) yielded a 130.39% increase in TTMP and a 271.57% increase in acetoin.

**TABLE 4:** The concentrations of TTMP and other metabolites produced by diploid strains.

**Strains^*a*^**	**TTMP (mg/L)**	**Acetoin (mg/L)**	**Diacetyl (mg/L)**	**2,3-Butanediol (mg/L)**
AY15	3.29 ± 0.11	581.13 ± 54.13	27.43 ± 1.36	1,825.34 ± 30.81
AY-B1	3.65 ± 0.12	721.02 ± 49.42*	29.14 ± 1.58	1,259.41 ± 30.02**
AY-SB1	7.58 ± 0.17**	2,159.34 ± 160.73**	37.32 ± 2.55**	620.55 ± 16.23**
AY-D1B2	4.48 ± 0.11	909.72 ± 96.89**	28.62 ± 1.5	1,868.59 ± 28.72
AY-SD1B2	9.47 ± 0.21**	2,893.96 ± 184.93**	35.52 ± 2.47**	508.12 ± 15.84**

^*a*^Diploid strain: AY-B1 (*BDH1* single-allele-deletion), AY-SB1 (*BDH1* double-allele-deletion), AY-D1B2 (*BDH1* single-allele-deletion and *BDH2* single-allele-overexpression), and AY-SD1B2 (*BDH1* double-allele-deletion and *BDH2* double-allele-overexpression). The strains were fermented by corn-semi-solid medium at 30°C for 4 days. Values are means ± standard deviations from at least three independent tests. Statistical significance is denoted as **P < 0.01 and *P < 0.05.

The diploid strain AY-D1B2 represents *BDH1* single-allele-deletion and *BDH2* single-allele-overexpression. The production of TTMP and acetoin by the AY-D1B2 strain was 4.48 and 909.73 mg/L, respectively. Strain AY-D1B2 yielded a 36.17% increase in TTMP and a 56.54% increase in acetoin. The diploid strain AY-SD1B2 was *BDH1* double-allele-deletion and *BDH2* double-allele-overexpression. The TTMP and acetoin content represented a 2.87- and 4.97-fold increase compared to strain AY15. As shown in Table. 3 and Supplementary Figure 1B, the fermentation performance of the AY-B1, AY-SB1, AY-D1B2, and AY-SD1B2 were similar to that of parental strain AY15. The production of acetoin and TTMP produced by AY-SD1B2 was the highest among the four diploid strains, while the double-allele-deletion of *BDH1* and the double-allele-overexpression of *BDH2* had the greatest impact on acetoin and TTMP production. In addition, regardless of whether this was the result of the deletion or overexpression of *BDH1* and *BDH2*, the content of acetoin was positively correlated with TTMP production.

Diploid strains of *S. cerevisiae* are widely used for fermentation in the food industry, including bread making and wine brewing. The diploid strain of *S. cerevisiae* is used as a stable source of fermentation in Baijiu brewing. To ensure the fermentation performance of the strain, it is necessary to hybridize the haploid strains into diploid strains. Diploid strains AY-SB1 (*BDH1* double-allele-deletion) and AY-SD1B2 (*BDH1* double-allele-deletion and *BDH2* double-allele-overexpression) were found to produce high levels of acetoin, which resulted in increased TTMP production. Moreover, compared with the original strain AY15, there were no significant differences regarding the growth curve, the fermentation performance, or the production of esters or higher alcohols. It is worth noting that the *BDH1* double-allele-deletion and *BDH2* double-allele-overexpression were the most efficient in terms of increased TTMP production. This study provides an important reference for the application genetic engineering to *S. cerevisiae* in order to increase the yield of TTMP in Baijiu and other alcoholic beverages, thereby improving their beneficial health attributes.

### Production of Ester and Higher Alcohol by Recombinant Diploid Strains

**FIGURE 5:**
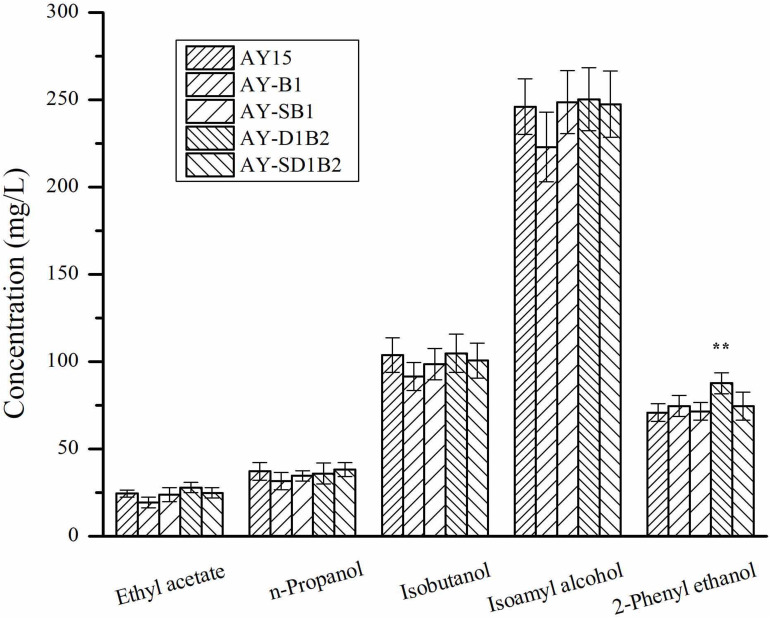
The content of esters and higher alcohols in diploid strains. Data represent the mean of three independent biological replicates. Error bars represent the SD of the average values. Statistical significance is denoted as **P < 0.01 and **P < 0.05.

To analyze the effects of the modification of *BDH1* and *BDH2* genes on the flavor substances, we detected the contents of esters and higher alcohols in diploid strains. The production of ester and higher alcohol was detected as a result of corn-semi-solid medium fermentation. As shown in Figure 5, the production of ethyl acetate by the AY15, AY-B1, AY-SB1, AY-D1B2, and AY-SD1B2 strains was 24.51, 19.44, 23.87, 27.98, and 24.92 mg/L, respectively. The AY-D1B2 strain showed a 23.71% decrease in the production of 2-phenylethanol. There were no significant changes in terms of the content of other higher alcohols in the fermentation samples of AY-B1, AY-SB1, AY-D1B2, and AY-SD1B2. These results indicated that the modification of *BDH1* and *BDH2* did not affect the flavor substance content produced by the diploid strain.

## Conclusion

2,3,5,6-Tetramethylpyrazine is a type of pyrazine that has a nutty aroma, and is a key flavor compound of Baijiu (Zhu et al., 2007). During Baijiu fermentation, acetoin is converted to TTMP spontaneously rather than as an enzymatic reaction, which is one of the reasons for the low TTMP content in Baijiu (Xiao et al., 2014; Zhang et al., 2019). In this study, we focus on regulating the expression levels of *BDH1* and *BDH2* genes to control the content of acetoin and TTMP. The results proved that knockout of *BDH1* or overexpression of *BDH2* can promote the synthesis of acetoin, and then increase the concentration of TTMP. In our research, knocking out *BDH1* and overexpressing *BDH2* in *S. cerevisiae* is the best strategy to increase the concentration of TTMP. It is important that the fermentation performance and the content of esters and higher alcohols produced by diploid strains (AY-B1, AY-SB1, AY-D1B2, and AY-SD1B2) with high TTMP production were not negatively affected. This study provides an important reference for the application genetic engineering to *S. cerevisiae* in order to increase the yield of TTMP in Baijiu and other alcoholic beverages, thereby improving their beneficial health attributes.

## Data Availability Statement

The original contributions presented in the study are included in the article/Supplementary Material, further inquiries can be directed to the corresponding author.

## Author Contributions

D-YC, D-GX, and C-YZ conceived and designed research. D-YC, Y-NW, and P-PF conducted experiments. L-CL, XL, and C-YZ analyzed data. D-YC and S-JC wrote the manuscript. D-GX, L-CL, and C-YZ revised and reviewed the manuscript. All authors read and approved the manuscript.

## Conflict of Interest

The authors declare that the research was conducted in the absence of any commercial or financial relationships that could be construed as a potential conflict of interest.

## Supplementary Material

Supplementary Figure 1 Growth curve of haploid strains and diploid strains.

Supplementary Table 1 Primers used in this study.

Supplementary Table 2 The recombinant fragments and corresponding PCR primers.
