# Supplementary material for: Increasing Yield of 2,3,5,6-Tetramethylpyrazine in Baijiu Through Saccharomyces cerevisiae Metabolic Engineering
Source: Front Microbiol. 2020 Nov 26;11:596306. doi: 10.3389/fmicb.2020.596306 (PMC7726194; doi:10.3389/fmicb.2020.596306)
Supplement: Supplementary Figure 1 — Growth curve of haploid strains and diploid strains. [file Image_1.pdf]

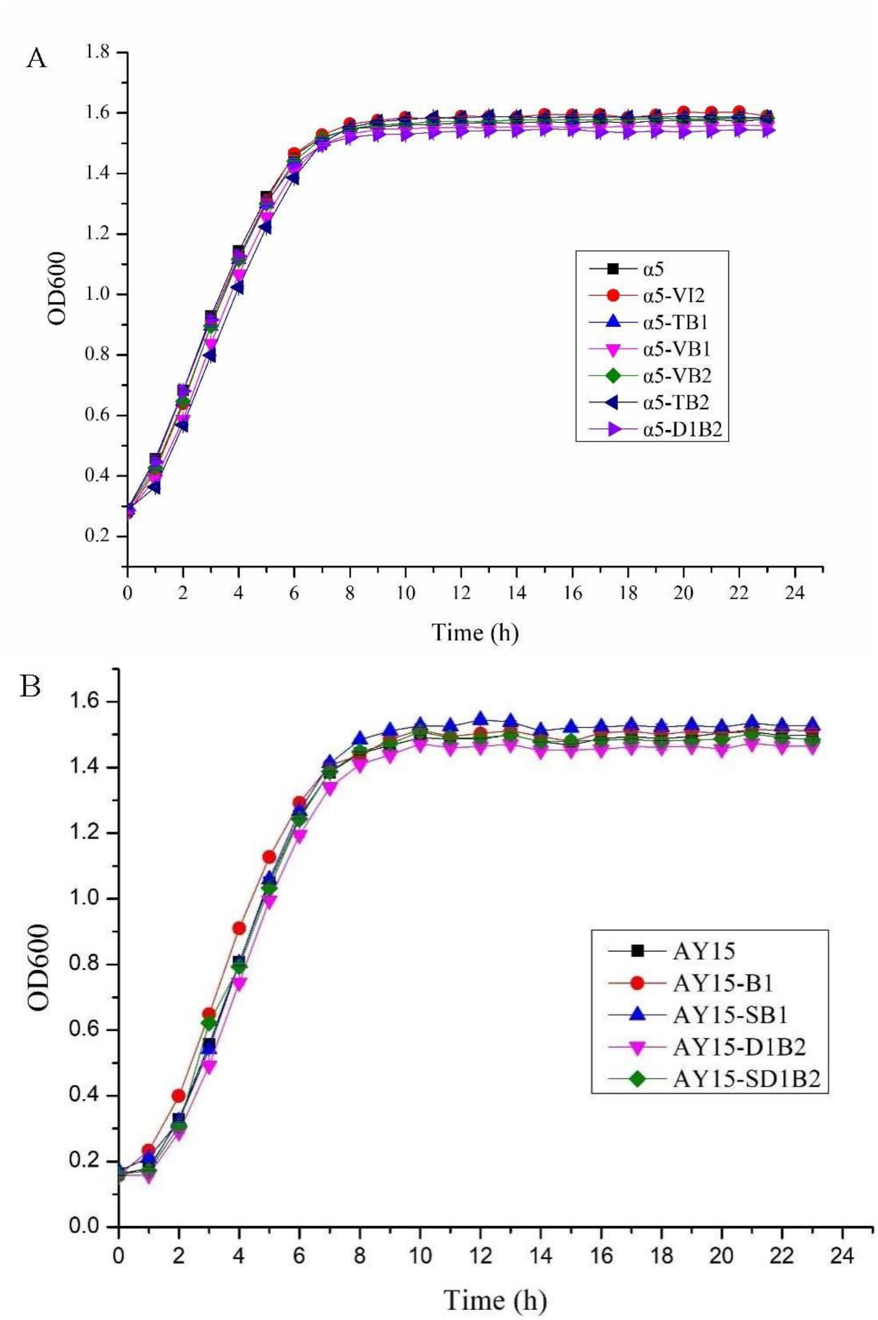

Supplementary Figure 1. Growth curve of haploid strains and diploid strains
A: The growth curves of α-type haploid recombinants and α5.
B: The growth curves of diploid recombinants and AY15.
Data represent the mean of three independent biological replicates. Error bars represent the SD of the average values.
